# Selenium in combination with a tomato lipid extract as a therapy for benign prostatic hyperplasia and its alterations in rats with induced BPH

**DOI:** 10.1111/jcmm.17903

**Published:** 2023-09-19

**Authors:** David Julian Arias‐Chávez, Patrick Mailloux‐Salinas, Jessica Ledesma Aparicio, Elihu Campos‐Pérez, Omar Noel Medina‐Campos, José Pedraza‐Chaverri, Guadalupe Bravo

**Affiliations:** ^1^ Departmento de Farmacobiología Centro de Investigación y de Estudios Avanzados del IPN, Sede Sur Mexico City Mexico; ^2^ Departamento de Patología, ISSSTE Hospital General Dra. Matilde Petra Montoya Lafragua Mexico City Mexico; ^3^ Departamento de Patología Hospital Ángeles Lindavista Mexico City Mexico; ^4^ Laboratorio F‐315, Departamento de Biología, Facultad de Química Universidad Nacional Autónoma de México Mexico City Mexico

**Keywords:** benign prostatic hyperplasia, dihydrotestosterone, finasteride, inflammation, oxidative stress, PSA, selenium, testosterone, tomato

## Abstract

Benign prostatic hyperplasia (BPH) is the most common adenoma in old men. Tomatoes are a rich source of bioactive compounds that, as well as selenium (Se), possess antioxidant and antiproliferative activity. The aim was to evaluate the therapeutic effect of Se in combination with a tomato extract in aged rats with BPH. Aged male Wistar rats were divided in the following groups (*n* = 10 rats/group): Control (C), BPH, BPH + Finasteride (BPH + F), BPH + Tomato Lipidic Extract (BPH + E), BPH + Selenium (BPH + S) and BPH plus E plus S (BPH + E + S). After 4 weeks of treatment, prostate weight, diuresis, antioxidants enzymes, prooxidants and inflammatory markers, growth factors and androgens were determined. BPH + E + S reduced prostate weight by 59.29% and inhibited growth by 99.35% compared to BPH + F which only decreased weight and inhibited growth by 15.31% and 57.54%, respectively. Prooxidant markers were higher with BPH + F (49.4% higher vs. BPH), but BPH + E + S decreased these markers (94.27% vs. BPH) and increased antioxidant activity. Finally, diuresis was higher with the BPH + E + S combination and markers of inflammation and growth factors were significantly lower with respect to BPH + F. Our findings provide a beneficial and protective therapeutic option of E + S directed against androgens, oxidative stress and inflammation that regulates cell proliferation in the prostate gland.

## 1 INTRODUCTION

The increase in prostate size causes bladder obstruction and physical compression of the urethra leading to lower urinary tract symptoms (LUTS).^1^, ^2^, ^3^ LUTS are the first cause of consultation in urology departments and are strongly associated with benign prostatic hyperplasia (BPH), the most frequent benign tumour in the elderly men >50 years of age.^3^, ^4^, ^5^

The physiopathology of BPH is unclear. However, aging, hormonal dysregulation, inflammation and oxidative stress (OS) are known to be strongly involved with this condition.^6^, ^7^, ^8^, ^9^ The binding of dihydrotestosterone (DHT) with the androgen receptor (AR) forms a complex that can attach to DNA to regulate genes responsible for producing growth factors associated to epithelial and stromal cell proliferation and increased of prostate‐specific antigen (PSA).^7^, ^8^ Local or systemic inflammation, which has been associated with OS, may be an important etiologic factor in the occurrence of LUTS associated with BPH.^9^, ^10^

Finasteride is the current first‐line pharmacological for BPH as it inhibits 5‐alpha reductase (5‐AR) preventing the conversion of testosterone to DHT which is involved in both normal and abnormal prostate growth.^11^, ^12^ Nevertheless, its use generates adverse effects like erectile dysfunction, decreased libido, gynecomastia and psychiatric disorders,^12^, ^13^ and although some therapeutic alternatives for BPH have been reported,^5^, ^14^, ^15^, ^16^, ^17^ the search of other treatments is relevant.

Selenium (Se) is an important trace element in health because participates in diverse processes as thyroid hormone metabolism, immunity, inflammation, apoptosis, cellular proliferation and antioxidant defence. Its deficiency has been associated to many disorders.^16^, ^18^, ^19^ Coadministration of Se with other compounds (lycopene) or plants extracts (*Serenoa repens*) has been found to have greater efficacy in reducing prostate size, inflammation and OS rather than individually.^14^, ^16^, ^20^ Moreover, tomato is a rich source of bioactive compounds such as minerals, polyphenols, vitamins and carotenes with antioxidant and anti‐inflammatory activity; they also induce the downregulation of the 5‐AR enzyme and androgen receptor (AR), thus reducing DHT‐induced cell proliferation.^21^, ^22^, ^23^, ^24^ The aim of this study was to evaluate the therapeutic effect of Se in combination with a tomato lipidic extract in comparison with finasteride in aged male rats.

## 2 MATERIALS AND METHODS

### 2.1 Reagents

Finasteride (TEALEP®) was purchased from Asofarma de México (Santa Fe, CDMX, Mexico). Testosterone enanthate (Testoprim‐D®) was obtained from Tocogino Laboratorios (Cuauhtémoc, CDMX, Mexico). Sodium selenite was purchased from Sigma‐Aldrich. All other reagents were reactive‐grade. Stock solutions and standard solutions were prepared daily to construct the calibration curves.

### 2.2 Experimental animals

Sixty male Wistar rats (3 months old) were obtained from Cinvestav‐IPN Pharmacobiology Department's animal facility. All animal procedures and protocols of the present research were approved by our Institutional Ethics Committee (CICUAL‐Cinvestav‐IPN) and followed the regulations established by the Mexican Official Standard for the Use and Welfare of Laboratory Animals (NOM‐062‐ZOO‐1999). Rats were housed individually in a controlled environment room with a 12‐h light–dark cycle (light from 7 to 19 h). All animals received LabDiet 5008® rat chow (Richmond, IN, USA) and had free access to water. After 11 months of maintenance, the aged animals (14 months old) were randomly into six groups (*n* = 10 rats/group), of which only the control (C) was not treated with testosterone enanthate to induce BPH. The other five groups were as follows: BPH; BPH + finasteride (B + F); BPH + tomato lipidic extract (BPH + E), BPH + selenium (B + S) and BPH + tomato lipidic extract + selenium (BPH + E + S).

### 2.3 BPH induction and treatments

To induce prostate growth by androgenic stimulation, the five groups of animals indicated above were injected with testosterone enanthate (10 mg/kg s.c; weekly) for 4 weeks. After this time each group of animals received the following treatments for 4 weeks: finasteride (5 mg/kg/day p.o.); tomato lipidic extract [5 mg/kg/day p.o. (with respect to lycopene concentration)], selenium (10 μg/kg/day p.o.) and a combination of tomato lipidic extract with selenium (5 mg/kg/day and 10 μg/kg/day p.o., respectively). Testosterone was administered during the 8‐week duration of the protocol.

### 2.4 Preparation of tomato extract

The tomato lipidic extract was prepared following the indications of the Mexican patent for the product No. 380295. Succinctly, tomato powder (saladette tomato variety) was mixed with corn oil at 60% (w/v). The mixture was sonicated (Branson® 3210) at 60 Hz for 1.5 h to perform the extraction in the oil by ultrasound‐induced cavitation effect. Lycopene concentration was evaluated spectrophotometrically. A 1:20 dilution of the extract was made and then placed in cells for spectrophotometry of 1 cm length and measured at 503 nm compared against the blank, a 1:20 dilution of corn oil (vehicle). The concentration of lycopene was calculated using the molar extinction coefficient of lycopene (17.2 × 10^−4^/M·cm) using the following equation: lycopene (mg/mL) = (Absorbance_503_/17.2 × 10^−4^/M·cm).

### 2.5 Diuresis/water consumption (bladder obstruction)

Weekly, the animals were placed in acrylic metabolic boxes with water ad libitum to collect urine for 12 h for 4 weeks. Subsequently, the amount of water consumed and the volume of urine were quantified.

### 2.6 Samples preparation

Once the treatment was completed, the animals were sacrificed by decapitation and truncal blood and prostates were obtained. Then, 100 mg of prostatic tissue was homogenized in 1 mL of PBS. The blood and homogenates were centrifuged at 10,062 *g* for 10 min and the supernatant was separated into aliquots and stored at −70°C for later use.

### 2.7 Anatomopathological analysis

The prostates were fixed in 10% formalin, dehydrated, and fixed in paraffin for histological analysis. The tissues were cut in 7‐μm slices, stained with haematoxylin–eosin and observed with an optical microscope to examine and evaluate changes in the micro‐architecture. Blind analysis was carried out in detail by an anatomopathologist. Morphometric measurements of the prostate glands were analysed using FIJI ImageJ 1.53j.

### 2.8 Oxidative damage/oxidant stress

Assays for malondialdehyde (MDA) and nitrites (${\mathrm{NO}}_{2}^{-}$) in prostate samples were performed as previously reported.^5^

### 2.9 Enzymatic antioxidant activity

Glutathione peroxidase (GPx) (Sigma‐Aldrich® catalog: CGP1), superoxide dismutase (SOD) (Sigma‐Aldrich® catalog: 19160) and catalase (CAT) (Sigma‐Aldrich® catalog: CAT100) activities in prostate samples were measured using the enzymatic colorimetric technique following the manufacturer's instructions.

### 2.10 Inflammatory markers and growth factors

IL‐1β (RayBio® catalog: ELR‐IL1b‐CL‐1), IL‐6 (RayBio® catalog: ELR‐IL6‐CL‐1) and TNF‐α (RayBio® catalog: ELR‐TNFa‐CL‐1), VEGF (MyBiosource® catalog: MBS760751), and F2GF (MyBiosource® Catalog: MBS459298) levels were determined using the ELISA technique following the manufacturer's instructions.

### 2.11 Dihydrotestosterone, testosterone, and PSA assay

DHT (MyBiosource® catalog: MBS265478), testosterone (CUSABIO® catalog: CSB‐E05100r) and PSA (MyBiosource® catalog: MBS704638) levels were measured using the ELISA technique following the manufacturer's instructions.

### 2.12 Statistical analyses

All the data were analysed using GraphPad Prism version 9.0 (GraphPad Software). Data are presented as mean ± SEM. The results obtained were analysed statistically to verify normality of the groups with the Shapiro–Wilkins, homogeneity of variances with Bartlett's test and one‐way analysis of variance (ANOVA) with Tukey's post hoc test for multiple comparisons. Values of *p* < 0.05 were considered significant.

## 3 RESULTS

### 3.1 Diuresis/water consumption (bladder obstruction)

**FIGURE 1:**
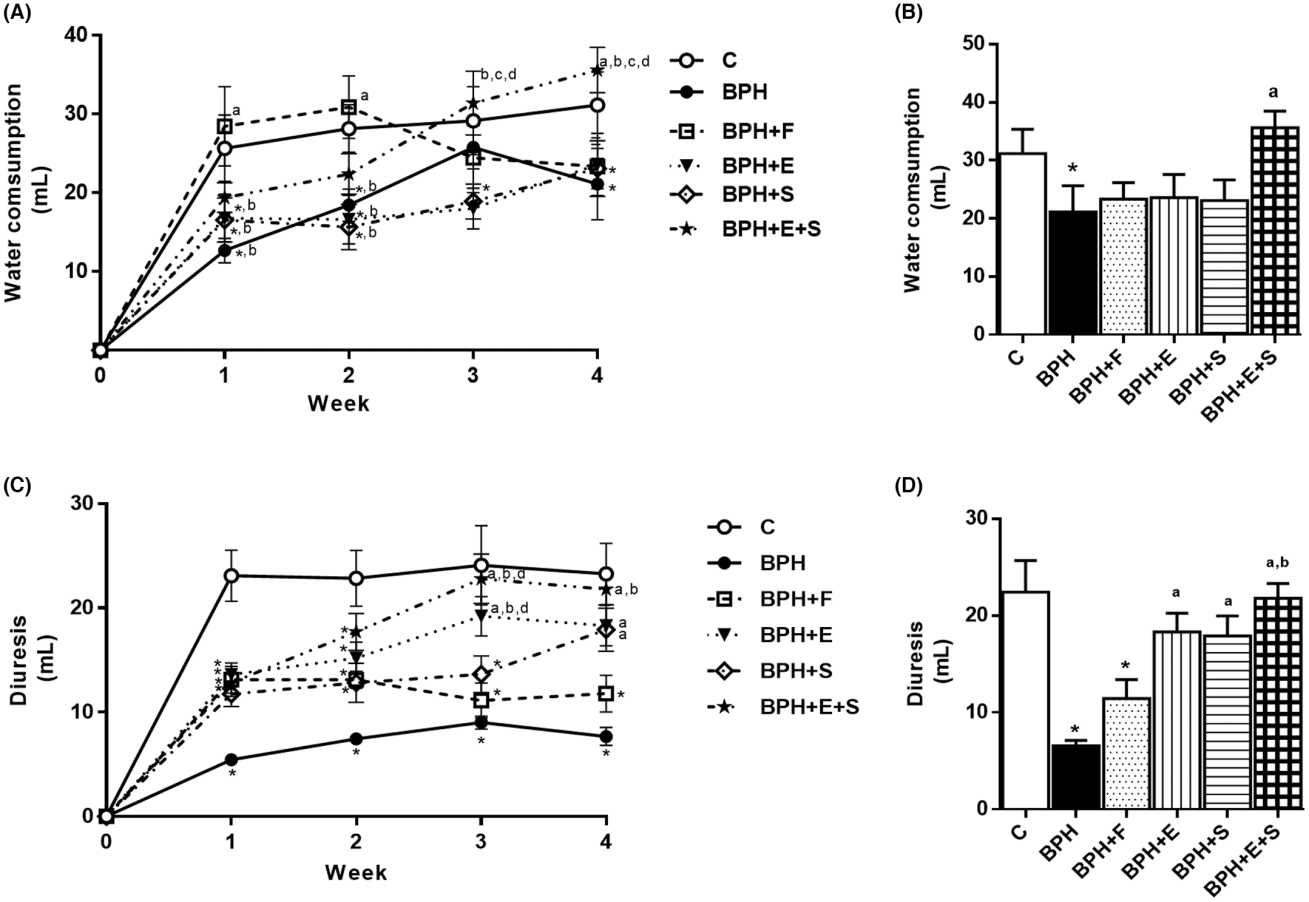
Diuresis/water consumption (bladder obstruction). (A) Time course of water consumption. (B) Water consumption at the end of treatments. (C) Time course of diuresis. (D) Diuresis at the end of treatment. C: control; BPH: benign prostatic hyperplasia; F: finasteride; E: tomato lipidic extract; S: selenium. Values represented as mean ± SEM ANOVA one way. Post hoc Tukey. *p < 0.05 versus C. ^a^ *p* < 0.05 versus BPH. ^b^ *p* < 0.05 versus F. *n* = 10. ^c^ *p* < 0.05 versus E. ^d^ *p* < 0.05 versus S.

During the 4 weeks of treatments, BPH group significantly consumed less water than C group (Figure 1A,B). It was observed that BPH + F group from second week onwards, consumed less water but it was not different from BPH group. BPH + E and BPH + S groups also decrease consumption but did not show differences versus BPH. Interestingly, BPH + E + S group, from second week, increased water consumption compared to BPH and did not present differences compared to C at the end of treatments. Regarding to diuresis it was significantly lower in BPH than C group from week 1 until the end of treatment. BPH + F group had differences with BPH, except for the third week. BPH + S and BPH + E groups had an increase in diuresis from the first week to the end of treatment with no difference between them but significantly greater than BPH group. BPH + E + S had the greatest increase in diuresis from the beginning of treatment with no difference with C group at the end of treatments. (Figure 1C,D). These data suggest that the combination of tomato extract with selenium decreases bladder obstruction and improves diuresis, which generates a decrease in LUTS.

### 3.2 Body, prostate and bladder changes

Group C did not differ from the BPH and BPH with treatment groups with respect to body weight. (Table 1). The prostate weight and prostatosomatic index of the BPH group presented a significant difference compared to C group with an increase 2.23‐fold. BPH + F group had a decrease of 15.31% prostate weight and 57.54% growth inhibition compared to BPH group, while BPH + E and BPH + S group decreased 36.93% and 32.35% prostate weight as well as 78.10% and 73.90% growth inhibition, respectively, compared to BPH group. However, BPH + E + S group decreased 59.29% and 99.35% prostate weight and growth inhibition, respectively (Table 1). On the other hand, the bladder was significantly larger in BPH group with respect to C group. In comparison with BPH group, BPH + F group decreased bladder weight, but it was significantly higher compared to BPH + E, BPH + S and BPH + E + S. Otherwise, BPH + E, BPH + S and BPH + E + S groups had no differences compared to C group. No differences were seen between BPH + E, BPH + S, BPH + E + S and C group (Table 1). These results indicate that testosterone plays a key role in prostatic growth and suggests that this growth generates a possible bladder obstruction causing and hence the increase in bladder weight in BPH group. In addition, the combination was able to inhibit prostate growth (99.35%) and enlargement (59.29%) more effectively and in less time than finasteride.

**TABLE 1:** Body, prostate, and bladder changes.

Group	Body weight (g)	Prostate weight (g)	Prostatosomatic index (%)	Decrease in prostate weight (%)	Growth inhibition (%)	Bladder weight (g)
C	649.8 ± 18.49	0.68 ± 0.04	0.11 ± 0.006	–	–	0.225 ± 0.007
BPH	602.1 ± 21.27	1.52 ± 0.03*	0.25 ± 0.008*	–	–	0.3 ± 0.01*
BPH+F	597.7 ± 24.69	1.29 ± 0.04*^,a^	0.22 ± 0.010*^,a^	15.31	57.54	0.281 ± 0.012*
BPH+E	602.9 ± 28.46	0.96 ± 0.05*^,a,b^	0.16 ± 0.007*^,a,b^	36.93^b^	78.10^b^	0.226 ± 0.008^a,b^
BPH+S	601.2 ± 15.80	1.03 ± 0.05*^,a,b^	0.17 ± 0.07*^,a,b^	32.53^b^	73.90^b^	0.228 ± 0.007^a,b^
BPH+E + S	613.8 ± 23.68	0.70 ± 0.06^a,b,c,d^	0.12 ± 0.007^a,b,c,d^	59.29^b,c,d^	99.35^b,c,d^	0.21 ± 0007^a,b^

*Note*: Values represented as mean ± SEM ANOVA one way. Post hoc Tukey. *p < 0.05 versus C. ^a^ *p* < 0.05 versus BPH. ^b^ *p* < 0.05 versus F. ^c^ *p* < 0.05 versus E. ^d^ *p* < 0.05 versus S. *n* = 10. Prostatosomatic index % (prostate weight/body weight*100).

Abbreviations: BPH, benign prostatic hyperplasia; C, control; E, tomato lipidic extract; F, finasteride; S, selenium.

### 3.3 Anatomopathological analysis

**FIGURE 2:**
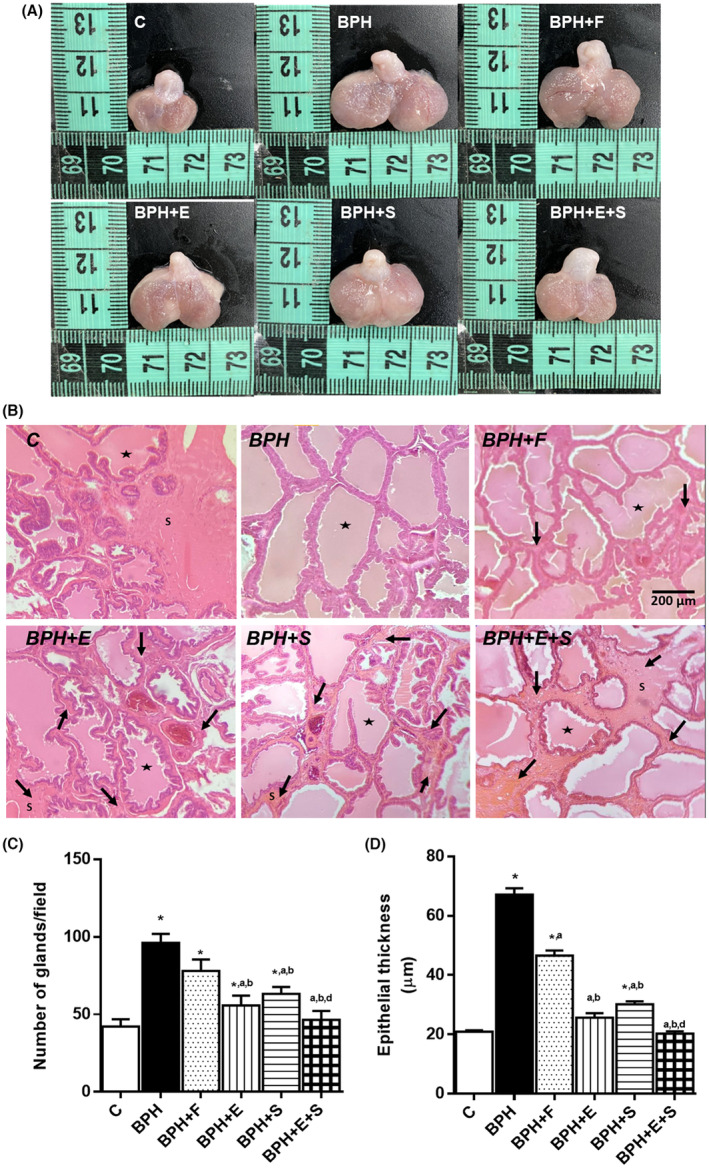
Anatomopathological analysis. (A) Photographs of prostate after treatment. (B) Staining of ventral prostate with haematoxylin–eosin. View 10×. (C) Number of glands/field. (D) Epithelial thickness. C: control; BPH: benign prostatic hyperplasia; F: finasteride; E: tomato lipidic extract; S: selenium. Values represented as mean ± SEM ANOVA one way. Post hoc Tukey. *p < 0.05 versus C. ^a^ *p* < 0.05 versus BPH. ^b^ *p* < 0.05 versus F. ^c^ *p* < 0.05 versus E. ^d^ *p* < 0.05 versus S. *n* = 10. ↓: indicate stroma (S). ★: prostate gland. Scale bars: 200 μm.

In group C, Figure 2A–D show the normal size and microarchitecture of the prostate glands without the presence of glandular hyperplasia, oedema and inflammatory infiltrate and with stroma around the glands. However, in group BPH, moderate to severe glandular hyperplasia and increased thickness of the epithelium, and size of the prostate, as well as stromal collapse with presence of oedema and inflammatory infiltrate were present. On the other hand, BPH + F group had a decrease in hyperplasia and thickness of the glandular epithelium, but with presence of oedema and inflammatory infiltrate and a slight stromal restructuring; finally BPH + E and BPH + S had a mild hyperplasia with predominance of no presence of glandular hyperplasia and stromal restructuring and decrease in the thickness of the epithelium, being BPH + E + S which presented a microarchitecture and size with great similarity with C. (Figure 2A–D). These results confirm the prostatic changes previously observed by the reversion and inhibition of cell proliferation in the group that received the combined treatment.

### 3.4 Prooxidant and antioxidant markers

**FIGURE 3:**
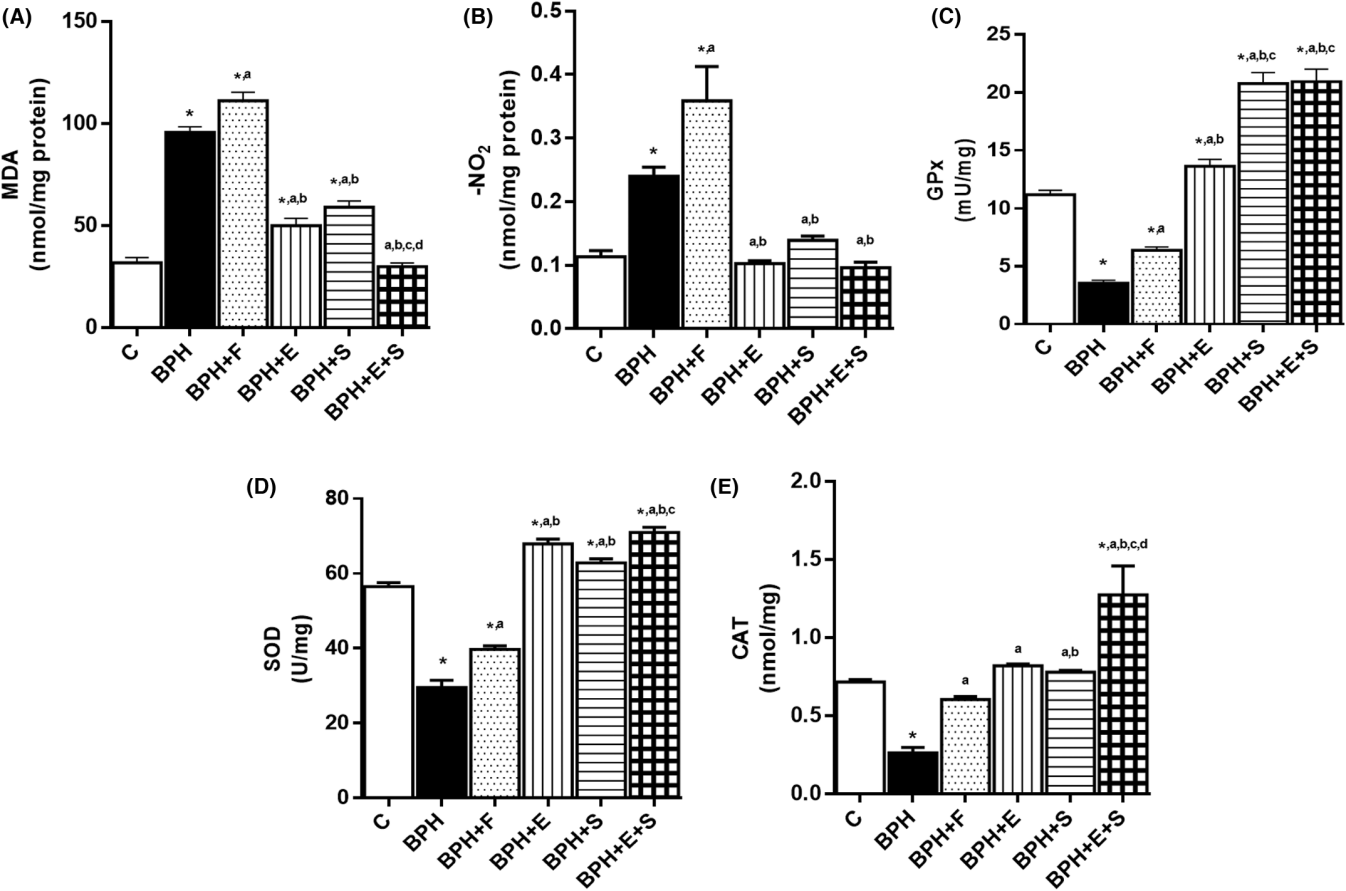
Prooxidants and antioxidant enzymes. (A) MDA. (B) ${\mathrm{NO}}_{2}^{-}$. (C) GPx. (D) SOD. (E) CAT. C: control; BPH: benign prostatic hyperplasia; F: finasteride; E: tomato lipidic extract; S: selenium. MDA: malondialdehyde. ${\mathrm{NO}}_{2}^{-}$: nitrites. GPx: glutathione peroxidase. SOD: superoxide dismutase. CAT: catalase. Values represented as mean ± SEM ANOVA one way. Post hoc Tukey. *p < 0.05 versus C. ^a^ *p* < 0.05 versus BPH. ^b^ *p* < 0.05 versus F. ^c^ *p* < 0.05 versus E. ^d^ *p* < 0.05 versus S. *n* = 10.

In the oxidative stress markers of the BPH group, there was a significant increase in MDA and ${\mathrm{NO}}_{2}^{-}$ with respect to the C group. However, BPH + F group was the one who had a significantly higher increase with respect to BPH in both MDA and ${\mathrm{NO}}_{2}^{-}$. BPH + E and BPH + S groups had a significant decrease compared to BPH group, with no differences between them BPH + E + S group did not present significant differences with respect to C group in both markers (Figure 3A,B). With respect to antioxidant enzymes, BPH group had a significant decrease versus C group in SOD, CAT and GPx. With respect to BPH + F group, it had an increase compared to BPH in all three enzymes. E group was higher compared to BPH and C in SOD and GPx but only with BPH group in CAT. This same trend was observed in S group, however, in GPx the increase was also higher compared to BPH + E group. Finally, BPH + E + S group had the highest increase in all three enzymes and particularly, in CAT, the levels were significantly higher compared to all groups. (Figure 3C–E). These results confirm the antioxidant activity of selenium and the extract and its potentiation when administered together.

### 3.5 Proinflammatory markers and growth factors

The proinflammatory cytokines IL‐1β, IL‐6 and TNF‐α had a significant increase in BPH compared to C group. BPH + F, BPH + E and BPH + S groups presented a decrease with respect to BPH, but with significant differences versus C, with BPH + E presenting the greatest decrease. On the other hand, in BPH + E + S group the decrease was greater without differences compared to C group (Table 2). On the other hand, in VEGF levels, BPH and BPH + F groups, had a significant increase compared to C group without differences between them while BPH + E, BPH + S and BPH + E + S presented a greater decrease without differences between them and C group (Table 2). Finally, for F2GF, BPH group was significant in comparison with C group, while BPH + F was not different from BPH group, BPH + E and BPH + S groups had a greater decrease with respect to BPH and BPH + F groups, but it was BPH + E + S group that did not present significant differences with respect to C group (Table 2).

**TABLE 2:** Proinflammatory markers and growth factors.

Group	Cytokines proinflammatory			Growth factors	
	IL‐1β (ng/mg)	IL‐6 (ng/mg)	TNF‐α (ng/mg)	VEGF (ng/mg)	F2GF (ng/mg)
C	68.38 ± 0.87	194.5 ± 5.74	190.9 ± 2.99	117.0 ± 2.13	130.4 ± 1.17
BPH	116.1 ± 2.33*	495.1 ± 12.24*	332.7 ± 10.04*	136.6 ± 1.03*	167.0 ± 1.76*
BPH+F	92.65 ± 1.66*^,a^	366.9 ± 17.01*^,a^	281.4 ± 6.58*^,a^	131.2 ± 1.07*^,a^	160.6 ± 2.02*
BPH+E	76.89 ± 1.51*^,a,b^	255.4 ± 4.92*^,a,b^	237.8 ± 4.24*^,a,b^	119.6 ± 1.02^a,b^	141.6 ± 1.31*^,a,b^
BPH+S	83.34 ± 1.03*^,a,b^	253.4 ± 6.83*^,a,b^	245.3 ± 2.53*^,a,b^	121.7 ± 0.97^a,b^	143.2 ± 1.54*^,a,b^
BPH+E + S	72.75 ± 1.56*^,a,b^	210.1 ± 5.64^a,b,c,d^	201.2 ± 4.54^a,b,c,d^	117.4 ± 1.95^a,b^	131.3 ± 1.38^a,b,c,d^

*Note*: Values represented as mean ± SEM ANOVA one way. Post hoc Tukey. *p < 0.05 versus C. ^a^ *p* < 0.05 versus BPH. ^b^ *p* < 0.05 versus F. ^c^ *p* < 0.05 versus E. ^d^ *p* < 0.05 versus S. *n* = 10.

Abbreviations: BPH, benign prostatic hyperplasia; C, control; E, tomato lipidic extract; F, finasteride; F2GF, fibroblast growth factor 2; IL‐1β, interleukin 1 beta; IL‐6, interleukin 6; S, selenium; TNF‐α, tumour necrosis factor alpha; VEGF, vascular endothelial growth factor.

### 3.6 Androgens and PSA levels

Testosterone at serum level increased significantly in all experimental groups compared to C group (Table 3). On the other hand, in prostatic testosterone levels BPH group had a significant increase compared to C group, while BPH + F and BPH + S groups decreased prostatic testosterone levels versus BPH group, BPH + E and BPH + E + S groups were not different from C group (Table 3). This same trend was observed in serum and prostatic DHT levels except for BPH + E and BPH + E + S groups that were also different from S group (Table 3). For the case of PSA, as well as DHT levels, BPH group was different from C group, while the groups that received treatment significantly decreased PSA levels, only BPH + E + S group was not different in compassion with C group (Table 3).

**TABLE 3:** Androgens and PSA levels.

Group	Testosterone		Dihydrotestosterone		PSA
	Serum (ng/mL)	Prostate (ng/mg)	Serum (pg/mL)	Prostate (ng/mL)	Serum (pg/mL)
C	0.066 ± 0.005	0.848 ± 0.036	1.704 ± 0.006	0.055 ± 0.003	7.977 ± 0.73
BPH	0.154 ± 0.017*	1.651 ± 0.053*	1.764 ± 0.004*	0.118 ± 0.004*	18.970 ± 0.85*
BPH+F	0.136 ± 0.007*	1.210 ± 0.035*^,a^	1.705 ± 0.004^a^	0.069 ± 0.002*^,a^	13.850 ± 0.36*^,a^
BPH+E	0.128 ± 0.005*	1.042 ± 0.048*^,a,b^	1.723 ± 0.004^a,b^	0.065 ± 0.002^a^	9.815 ± 0.48*^,a,b^
BPH+S	0.127 ± 0.007*	1.096 ± 0.062*^,a^	1.745 ± 0.002*^,a,b,c^	0.073 ± 0.001*^,a^	11.180 ± 0.37*^,a,b,c^
BPH+E + S	0.128 ± 0.006*	0.929 ± 0.025^a,b^	1.720 ± 0.006^a,d^	0.063 ± 0.002^a^	8.656 ± 0.62^a,b,c^

*Note*: Values represented as mean ± SEM ANOVA one way. Post hoc Tukey. *p < 0.05 versus C. ^a^ *p* < 0.05 versus BPH. ^b^ *p* < 0.05 versus F. ^c^ *p* < 0.05 versus E. ^d^ *p* < 0.05 versus S. *n* = 10.

Abbreviations: BPH, benign prostatic hyperplasia; C, control; DHT, dihydrotestosterone; E, tomato lipidic extract; F, finasteride; PSA, prostate‐specific antigen; S, selenium.

## 4 DISCUSSION

The aetiology and origin of BPH are regulated by different factors such as aging, androgens, inflammation and OS, which favour the development of LUTS and affect the quality of life of patients.^5^, ^9^, ^25^, ^26^ The prostate is an androgen‐dependent tissue in which DHT levels are 10–20 times higher than those of testosterone, and the levels of these androgens are increased in BPH.^27^ Exogenous administration of testosterone in experimental animal models had a significant increase of weight prostate.^14^, ^15^, ^16^ These data directly demonstrate that exogenous androgen stimulation significantly increases the weight of the prostate gland, and in severe cases, completely obstructs the urethra generating LUTS.^28^, ^29^ Urinary retention due to BPH originates inflammation and urinary tract infections due to prolonged storage of urine. As an adaptive response to increased urine volume, the bladder undergoes morphological changes generating bladder hyperactivity.^9^, ^28^, ^29^ This would explain why the BPH group increased bladder weight, decreased diuresis and increased inflammatory markers, while the treatments improved urinary retention due to decreased prostate volume.

The prostate is an immunocompetent organ populated by T lymphocytes; however, physiological factors such as aging and OS, as well as external factors such as lifestyle and exogenous testosterone have been associated with increased inflammatory infiltrate of lymphocytes and macrophages, as well as proinflammatory cytokines.^26^, ^30^ The degree of inflammation that occurs in BPH has been correlated with increased AR expression, DHT levels, urination and acute urinary retention.^9^, ^26^ On the other hand, DHT can up‐regulate the expression of the inflammatory factor TNF‐α by stimulating AR in macrophages and increasing inflammation, while inflammation is able to alter the cell proliferation/death state and generate overexpression of AR and induce activation of growth factor signalling pathways, responsible for prostatic cell proliferation and PSA.^7^ DHT has been reported to increase AR signalling 10‐fold compared to testosterone, in addition to an overexpression of 5‐AR in BPH.^7^ Thus, our data are consistent in the critical role of androgens in increasing inflammation and with it, OS, growth factors and PSA, as an indicator of increased prostate volume, as well as increased LUTS, as measured by decreased diuresis.

Se is the most required micronutrient by humans due to its participation in various metabolic and cellular functions.^31^ This chemical element forms organic compounds due to its strong binding to amino acids and proteins formation, generating selenoproteins of great biological importance in health such as GSH, GPx and thioredoxin reductases due to their antioxidant, anti‐inflammatory, anti‐proliferative and pro‐apoptotic activity.^16^, ^18^, ^19^, ^32^, ^33^, ^34^ Data from BPH + S group indicate that this micronutrient inhibits prostate growth, decreased OS and cell proliferation. The decrease of inflammation and androgens could be explained by their antiproliferative and pro‐apoptotic effects, decreasing the number of cells and, therefore, the expression of AR. However, further studies are required to know the possible mechanism of action responsible for this effect.

On the other hand, tomato is a rich source of bioactive compounds like vitamins (B, C and E), minerals (P, K, Zn, Cu, Mn), carotenoids, (lycopene α and β‐cartene) and phenolics compounds (quercetin, myricetin and kaempferol).^21^, ^23^ It has been reported that whole tomato consumption has a synergistic action due to all its nutraceutical compounds compared to lycopene consumption alone.^35^, ^36^ Lycopene, flavonoids such as flavonols, flavanones and flavones and other compounds present in tomato have been observed to decrease prooxidant markers, inflammation, PSA, DHT, increase antioxidant activity that inhibits 5‐AR and IGF‐1 signal transduction and androgen‐mediated signalling by downregulating AR, and have been reported to decrease prostate size and LUTS and increased urinary flow in both in vitro and in vivo studies.^12^, ^21^, ^24^, ^37^, ^38^, ^39^ The above‐mentioned bioactive compounds are present in different quantities in seeds, peel and pulp of tomato.^23^, ^36^ Lycopene and other compounds are present in greater quantities in skin and seeds, but not in pulp, and generally, the consumption of tomato through purees and tomato‐based preparations without seeds and peel provides a low concentration of these compounds of high biological value.^23^, ^36^ Our extract concentrates the main compounds of tomato and allows a better absorption due to its lipidic origin, having the advantage of not consuming high amounts of tomato and on the contrary small amounts with a therapeutic effect able to block and/or inhibit the main pathways that lead to BPH. According to these results, the administration of whole tomato extract had a greater effect in reversing hyperplasia, inflammation and OS, as well as PSA, androgens, growth factors and increased diuresis indicating that it can decrease LUTS by decreasing prostatic volume.

DHT has been the therapeutic target in recent decades because of its key role in the aetiology of BPH. As a first‐line hormonal treatment, finasteride, a 5‐AR inhibitor, suppresses the conversion of testosterone to DHT and can reduce prostate size by as much as 25%–30% with prolonged use over a period of 3–12 months.^12^ For this reason, it is usually administered in combination with an alpha‐adrenergic blocker to reduce LUTS, so that finasteride can have the desired therapeutic effect.^12^ However, by preventing DHT conversion, the overactivation of the androgen receptor helps to decrease inflammation and cell proliferation induced by growth factors activated by the DHT‐AR complex. Our results showed that finasteride increases MDA and ${\mathrm{NO}}_{2}^{-}$ levels above values of the BPH group. This could be related to one of its main adverse effects, which is erectile dysfunction, as free radicals rapidly sequester the nitric oxide responsible for vasodilation and, therefore, erection.^13^ Post‐finasteride syndrome has been reported due to the use of this drug during and after treatment for long time, which generates different adverse effects such as gynecomastia, loss or reduction of libido, ejaculatory dysfunction, insomnia and psychiatric illnesses and even suicidal ideation.^12^, ^13^

Nevertheless, unlike the therapeutic effects of Se and tomato extract, finasteride has unique mechanism of action and, in addition, the ability to generate oxidative stress according to our results. The aim of this work was to determine the therapeutic effect of Se in combination with a whole tomato extract on BPH. Although individually we observed a therapeutic effect of Se and tomato extract, our results indicate a greater effect by decreasing prostate weight and size and inhibiting growth almost completely when administered together. In addition, the antioxidant, anti‐inflammatory, antiproliferative and androgen modulating effects were greater in the combination than in the individual treatments. Our results are in agreement with previous reports, in which the combination of Se with lycopene and *S. repens* extract had a greater effect in decreasing prostate gland weight.^14^, ^15^, ^16^ Nonetheless, adverse effects have been reported for the use of *S. repens* like those reported for finasteride such as erectile dysfunction.^40^ Aging naturally increases OS, which is related to the pathophysiology of BPH. The accumulated production of reactive oxygen species due to endogenous or exogenous causes, like testosterone, plays a determinant role in diseases such as BPH.^5^, ^24^ The antioxidant effect of Se and the tomato extract together would have a positive effect not only on BPH but also in elderly subjects, improving their quality of life compared to finasteride, which would probably worsen OS.

Finally, this study demonstrates a greater safe and effective therapeutic effect of Se in combination with whole tomato lipidic extract on the aetiology of BPH through different mechanisms. The enhanced therapeutic effect of this combination may be an excellent therapeutic alternative in elderly patients with persistent BPH and LUTS.

## AUTHOR CONTRIBUTIONS

**David Julian Arias‐Chávez:** Conceptualization (equal); data curation (equal); formal analysis (equal); methodology (equal); supervision (equal); writing – original draft (equal); writing – review and editing (equal). **Patrick Mailloux‐Salinas:** Formal analysis (equal); methodology (equal); supervision (equal). **Jessica Ledesma‐Aparicio:** Methodology (equal); writing – original draft (equal). **Elihu Campos‐Pérez:** Methodology (equal); supervision (equal); visualization (equal). **Omar Noel Medina‐Campos:** Methodology (equal); writing – review and editing (equal). **José Pedraza‐Chaverri:** Investigation (equal); methodology (equal); writing – review and editing (equal). **Guadalupe Bravo:** Conceptualization (equal); investigation (equal); project administration (equal); writing – review and editing (equal).

## CONFLICT OF INTEREST STATEMENT

There is no conflict of interest that could be perceived as prejudicing the impartiality of the research reported.

## Data Availability

The datasets generated in the current study are available from the corresponding author on reasonable request.

## References

[1] Moul S , McVary KT . Lower urinary tract symptoms, obesity and the metabolic syndrome. Curr Opin Urol. 2010;20(1):7‐12. doi:10.1097/MOU.0b013e3283336f3f 19904208

[2] Blankstein U , Van Asseldonk B , Elterman DS . BPH update: medical versus interventional management. Can J Urol. 2016;23(Suppl 1):10‐15. PMID: 26924590. 26924590

[3] Parsons JK . Benign prostatic hyperplasia and male lower urinary tract symptoms: epidemiology and risk factors. Curr Bladder Dysfunct Rep. 2010;5(4):212‐218. doi:10.1007/s11884-010-0067-2 21475707 PMC3061630

[4] Chughtai B , Forde JC , Thomas DD , et al. Benign prostatic hyperplasia. Nat Rev Dis Primers. 2016;5(2):16031. doi:10.1038/nrdp.2016.31 27147135

[5] Colado‐Velázquez JIII , Mailloux‐Salinas P , Arias‐Chávez DJ , et al. Lipidic extract of whole tomato reduces hyperplasia, oxidative stress and inflammation on testosterone‐induced BPH in obese rats. Int Urol Nephrol. 2023;55(3):529‐539. doi:10.1007/s11255-022-03383-2 36464759

[6] Wen S , Chang HC , Tian J , Shang Z , Niu Y , Chang C . Stromal androgen receptor roles in the development of normal prostate, benign prostate hyperplasia, and prostate cancer. Am J Pathol. 2015;185(2):293‐301. doi:10.1016/j.ajpath.2014.10.012 25432062 PMC4305176

[7] Vickman RE , Franco OE , Moline DC , Vander Griend DJ , Thumbikat P , Hayward SW . The role of the androgen receptor in prostate development and benign prostatic hyperplasia: a review. Asian J Urol. 2020;7(3):191‐202. doi:10.1016/j.ajur.2019.10.003 32742923 PMC7385520

[8] D'Amico R , Genovese T , Cordaro M , et al. Palmitoylethanolamide/Baicalein regulates the androgen receptor signaling and NF‐κB/Nrf2 pathways in benign prostatic hyperplasia. Antioxidants. 2021;10(7):1014. doi:10.3390/antiox10071014 34202665 PMC8300753

[9] Lloyd GL , Marks JM , Ricke WA . Benign prostatic hyperplasia and lower urinary tract symptoms: what is the role and significance of inflammation? Curr Urol Rep. 2019;20(9):54. doi:10.1007/s11934-019-0917-1 31377881 PMC7339114

[10] Hung SF , Chung SD , Kuo HC . Increased serum C‐reactive protein level is associated with increased storage lower urinary tract symptoms in men with benign prostatic hyperplasia. PloS One. 2014;9(1):e85588. doi:10.1371/journal.pone.0085588 24454896 PMC3893218

[11] Chitturi S , Farrell GC . Chapter 33: adverse effects of hormones and hormone antagonists on the liver. Drug‐induced Liver Disease. Vol 1. 3rd ed. Elsevier; 2013:605‐619. doi:10.1016/B978-0-12-387817-5.00033-9

[12] Langan RC . Benign prostatic hyperplasia. Primary Care: Clinics in Office Practice. 2019;46(2):223‐232. doi:10.1016/j.pop.2019.02.003 31030823

[13] Traish AM . Post‐finasteride syndrome: a surmountable challenge for clinicians. Fertil Steril. 2020;113(1):21‐50. doi:10.1016/j.fertnstert.2019.11.030 32033719

[14] Altavilla D , Bitto A , Polito F , et al. The combination of *Serenoa repens*, selenium and lycopene is more effective than *Serenoa repens* alone to prevent hormone dependent prostatic growth. J Urol. 2011;186(4):1524‐1529. doi:10.1016/j.juro.2011.05.049 21855911

[15] Colado‐Velázquez J III , Mailloux‐Salinas P , Medina‐Contreras J , Cruz‐Robles D , Bravo G . Effect of *Serenoa repens* on oxidative stress, inflammatory and growth factors in obese wistar rats with benign prostatic hyperplasia. Phytother Res. 2015;29(10):1525‐1531. doi:10.1002/ptr.5406 26104840

[16] Minutoli L , Bitto A , Squadrito F , et al. *Serenoa repens*, lycopene and selenium: a triple therapeutic approach to manage benign prostatic hyperplasia. Curr Med Chem. 2013;20(10):1306‐1312. doi:10.2174/0929867311320100007 23432584

[17] Farshid MA , Fazeli M , Shomali T , Nazifi S , Namazi F . Protective effect of black mulberry (*Morus nigra* L.) fruit hydroalcoholic extract against testosterone‐induced benign prostatic hyperplasia in rats. Int J Androl. 2021;19(1):53‐61. doi:10.1016/j.androl.2019.09.003 31899187

[18] Bodnar M , Konieczka P , Namiesnik J . The properties, functions, and use of selenium compounds in living organisms. J Environ Sci Health. 2012;30(3):225‐252. doi:10.1080/10590501.2012.705164 22970720

[19] Ullah H , Liu G , Yousaf B , et al. Developmental selenium exposure and health risk in daily foodstuffs: a systematic review and meta‐analysis. Ecotoxicol Environ Saf. 2018;149:291‐306. doi:10.1016/j.ecoenv.2017.11.056 29268101

[20] Morgia G , Cimino S , Favilla V , et al. Effects of *Serenoa repens*, selenium and lycopene (Profluss®) on chronic inflammation associated with benign prostatic hyperplasia: results of "FLOG" (Flogosis and Profluss in Prostatic and Genital Disease), a multicentre Italian study. Int Braz J Urol. 2013;39(2):214‐221. doi:10.1590/S1677-5538.IBJU.2013.02.10 23683667

[21] Cicero AFG , Allkanjari O , Busetto GM , et al. Nutraceutical treatment and prevention of benign prostatic hyperplasia and prostate cancer. Arch Ital Urol Androl. 2019;91(3):139‐152. doi:10.4081/aiua.2019.3.139 31577095

[22] Jena AK , Vasisht K , Sharma N , Kaur R , Dhingra MS , Karan M . Amelioration of testosterone induced benign prostatic hyperplasia by *Prunus* species. Journal of Ethnopharmacoly. 2016;190:33‐45. doi:10.1016/j.jep.2016.05.052 27235020

[23] Kumar M , Tomar M , Bhuyan DJ , et al. Tomato (*Solanum lycopersicum* L.) seed: a review on bioactives and biomedical activities. Biomed Pharmacother. 2021;142:112018. doi:10.1016/j.biopha.2021.112018 34449317

[24] Zhang X , Wang Q , Neil B , Chen X . Effect of lycopene on androgen receptor and prostate‐specific antigen velocity. Chin Med J (Engl). 2019;123(16):2231‐2236. 20819671

[25] Al‐Barzinj RMGT . Estimation levels of prostate‐specific antigen, interleukin‐8, oxidative stress and some inflammatory markers in sera of benign prostatic hyperplasia patients who have smoking habits as a risk factor. Cell Mol Biol. 2020;66(7):124‐130. doi:10.14715/cmb/2020.66.7.19 33287932

[26] Tong Y , Zhou RY . Review of the roles and interaction of androgen and inflammation in benign prostatic hyperplasia. Mediators Inflamm. 2020;2020(28):7958316. doi:10.1155/2020/7958316 33192175 PMC7641707

[27] Pejčić T , Tosti T , Tešić Ž , et al. Testosterone and dihydrotestosterone levels in the transition zone correlate with prostate volume. Prostate. 2017;77(10):1082‐1092. doi:10.1002/pros.23365 28594074

[28] Devlin CM , Simms MS , Maitland NJ . Benign prostatic hyperplasia – what do we know? BJU Int. 2021;127(4):389‐399. doi:10.1111/bju.15229 32893964

[29] Espinosa‐Juárez JV , Colado‐Velázquez JI , Mailloux‐Salinas P , et al. Beneficial effects of lipidic extracts of saladette tomato pomace and *Serenoa repens* on prostate and bladder health in obese male wistar rats. J Sci Food Agric. 2017;97(13):4451‐4458. doi:10.1002/jsfa.8308 28276068

[30] Tyagi P , Wang Z , Yoshimura N . Urinary biomarkers and benign prostatic hyperplasia. Current Bladder Dysfunction Reports. 2019;14:31‐40. doi:10.1007/s11884-019-00504-z

[31] Hariharan S , Dharmaraj S . Selenium and selenoproteins: it's role in regulation of inflammation. Inflammopharmacology. 2020;28(3):667‐695. doi:10.1007/s10787-020-00690-x 32144521 PMC7222958

[32] Azimi H , Khakshur AA , Aghdasi I , Fallah‐Tafti M , Abdollahi M . A review of animal and human studies for management of benign prostatic hyperplasia with natural products: perspective of new pharmacological agents. Inflamm Allergy Drug Targets. 2012;11(3):207‐221. doi:10.2174/187152812800392715 22512478

[33] Lazard M , Dauplais M , Blanquet S , Plateau P . Recent advances in the mechanism of selenoamino acids toxicity in eukaryotic cells. Biomol Concepts. 2017;8(2):93‐104. doi:10.1515/bmc-2017-0007 28574376

[34] Plateau P , Saveanu C , Lestini R , et al. Exposure to selenomethionine causes selenocysteine misincorporation and protein aggregation in *Saccharomyces cerevisiae* . Sci Rep. 2017;7:44761. doi:10.1038/srep44761 28303947 PMC5355996

[35] Li N , Wu X , Zhuang W , et al. Tomato and lycopene and multiple health outcomes: umbrella review. Food Chem. 2021;1(343):128396. doi:10.1016/j.foodchem.2020.128396 33131949

[36] Romano R , De Luca L , Manzo N , Pizzolongo F , Aiello A . A new type of tomato puree with high content of bioactive compounds from 100% whole fruit. J Food Sci. 2020;85(10):3264‐3272. doi:10.1111/1750-3841.15423 32885436

[37] Silva SA , Gobbo MG , Pinto‐Fochi ME , et al. Prostate hyperplasia caused by long‐term obesity is characterized by high deposition of extracellular matrix and increased content of MMP‐9 and VEGF. Int J Exp Pathol. 2015;96(1):21‐30. doi:10.1111/iep.12107 25529509 PMC4352349

[38] Wang H , Leung LK . The carotenoid lycopene differentially regulates phase I and II enzymes in dimethylbenz [a] anthracene‐induced MCF‐7 cells. Nutrition. 2010;26(11–12):1181‐1187. doi:10.1016/j.nut.2009.11.013 20400267

[39] Nandy PR , Saha S . Association between components of metabolic syndrome and prostatic enlargement: an Indian perspective. Med J Armed Forces India. 2016;72(4):350‐355. doi:10.1016/j.mjafi.2016.07.005 27843182 PMC5099443

[40] Gallo E , Maggini V , Lombardi N , et al. *Serenoa repens* induced erectile dysfunction: underdiagnosis and phytovigilance. Br J Clin Pharmacol. 2022;88(5):2441‐2443. doi:10.1111/bcp.15129 34747053

